# Predictive modelling of Parkinson’s disease progression based on RNA-Sequence with densely connected deep recurrent neural networks

**DOI:** 10.1038/s41598-022-25454-1

**Published:** 2022-12-12

**Authors:** Siraj Ahmed, Majid Komeili, Jeongwon Park

**Affiliations:** 1 School of Electrical Engineering and Computer Science, University of Ottawa, Ottawa, ON K1N 6N5 Canada; 2 School of Computer Science, Carleton University, Ottawa, ON K1S 5B6 Canada; 3 Department of Electrical and Biomedical Engineering, University of Nevada, Reno, NV 89557 USA

**Keywords:** Computational biology and bioinformatics, Machine learning, Biotechnology, Genomics, Transcriptomics

## Abstract

The advent of recent high throughput sequencing technologies resulted in unexplored big data of genomics and transcriptomics that might help to answer various research questions in Parkinson’s disease (PD) progression. While the literature has revealed various predictive models that use longitudinal clinical data for disease progression, there is no predictive model based on RNA-Sequence data of PD patients. This study investigates how to predict the PD Progression for a patient’s next medical visit by capturing longitudinal temporal patterns in the RNA-Seq data. Data provided by Parkinson Progression Marker Initiative (PPMI) includes 423 PD patients without revealing any race, sex, or age information with a variable number of visits and 34,682 predictor variables for 4 years. We propose a predictive model based on deep Recurrent Neural Network (RNN) with the addition of dense connections and batch normalization into RNN layers. The results show that the proposed architecture can predict PD progression from high dimensional RNA-seq data with a Root Mean Square Error (RMSE) of 6.0 and a rank-order correlation of (r = 0.83, *p* < 0.0001) between the predicted and actual disease status of PD.

## Introduction

Parkinson’s Disease (PD) is a degenerative disorder that progresses over time affecting the central nervous system. PD affects 7–10 million people worldwide and is clinically characterized by a decline in both motor and non-motor abilities. The disease is highly idiopathic and currently, there is no cure. However, the symptoms can be managed to improve the quality of life^1^. Although the symptoms are the same across all PD patients, the disease progresses differently across different patients. For instance, few patients follow the fastest trajectory of disease progression and their condition worsens quickly. On the other hand, few patients follow a slow trajectory. Such heterogeneity in PD patients hinders the practitioners from prescribing appropriate treatment. Besides, such heterogeneity makes a clinical trial of disease-modifying therapies challenging as we now need a larger number of subjects to be enrolled which makes the process expensive and time-consuming^2^. Therefore, there is an unmet need for a prognostic tool to help the practitioners know beforehand if the newly diagnosed PD patient will progress quickly in the disease or has a slow progression rate. This will help support their decision to refer the patient to a larger, more experienced center for care very early on.

Machine learning algorithms have been applied to classify Parkinson's Disease (PD) from healthy subjects and to predict the disease progression of PD patients^3–8^. In the past, studies have investigated how clinical features data such as speech signals^4^, brain images and DaTscans^5^, gait sensor data^6^, handwriting^7^, sleep behavior disorders, and olfactory loss^8^ that capture PD clinical features have been used to predict disease progression in Parkinson disease. In addition to the clinical features data, we have diverse “Omics” data such as genomics, epigenomics, transcriptomics, proteomics, metabolomics, microbiomics that have the potential to provide a global view of the complex biological processes related to human diseases^9,10^. Transcriptomic study such as RNA-Sequence which is relatively newer omics data examines both qualitative and quantitative levels of RNA levels^10–12^. Deep Learning (DL) techniques are now often employed to improve prediction accuracy in comparison to other conventional machine learning techniques^3–8,10–12^. Eliza Courtney et al.^12^ investigated the usefulness of RNA-Sequence data in the research of neurodegenerative diseases such as Alzheimer’s disease, Parkinson’s disease, and Huntington’s disease. We know that neurodegenerative diseases are characterized by a significant change in gene expression levels and splicing patterns that occur before the onset and during the progression of the disease^1–8,10,12^. Hence, the authors called for a need to investigate RNA-Sequence data in these diseases as RNA-Sequence has the advantage of digital expression profiling and can identify alternative splicing patterns that have become a major focus of degenerative disease research^12^. It is important to predict disease severity or progression accurately in Parkinson's Disease. The usefulness of transcriptome data, such as RNA-Sequence, for the classification of Parkinson's Disease, however, has not been thoroughly studied. For disease prediction problems, the traditional machine learning algorithms predict the disease status by aggregating the longitudinal features rather than leveraging through the temporal patterns^13^. To address this problem of capturing patterns from longitudinal data, Wang et al.^13^ aimed to employ a two-layered RNN network of Long Short Term Memory (LSTM) to predict Alzheimer’s Disease (AD) status for a patient based on the historical “clinical” information of patients such as demographic data, health history, physical examination, etc. in a classification framework. However, for Parkinson's disease progression, we believe there is no study that leverages the longitudinal temporal “transcriptomic” information of historical visits to predict the future disease status in a regression framework. In this study, we explored whether artificial neural networks, particularly RNN, can forecast the pace of Parkinson's disease progression in the early stages of the condition. The result of this study may contribute to identify an RNN architecture that will be suitable for learning the longitudinal temporal patterns in high dimensional RNA-Sequencing data.

## Materials and methods

### Dataset description, analysis and pre-processing

The disease progression of Parkinson's disease that is well represented by the Movement Disorder Society-sponsored unified Parkinson’s disease rating scale (MDS-UPDRS)^14^ reflects the motor symptoms and clinometric properties of PD on a scale of 0 to 272 with 0 being normal and 272 being severe motor and non-motor decline. We aim to predict this MDS-UPDRS score of a patient for the immediate future hospital visit by considering the RNA sequence data of multiple previous visits. The datasets for this study were provided by PPMI^15^, which is sponsored by The Michael J. Fox Foundation. The PPMI Data Repository contains de-identified clinical, imaging, and omics data gathered as part of the PPMI project, an ongoing longitudinal investigation that was started in 2010 with the goal of locating biomarkers for the progression of Parkinson's disease. The PPMI has done a longitudinal study on 423 PD subjects who were drug naïve (i.e., not much treated with dopaminergic medications) and who have been diagnosed with PD for a period of less than 2 years at the time of enrollment. As we aim to build a disease progression tool for early diagnosed PD patients, the subjects being drug naïve is hypothesized to be beneficial. The dataset access and methods for the study were approved by the PPMI. All methods were performed in accordance with the relevant guidelines and regulations by the PPMI. Informed consent was obtained from all subjects and/or their legal guardians from the PPMI without revealing any race, sex, or age information.

The PPMI subjects were followed up for a period of 4 years from the date of enrollment and the clinical data was collected at uniform intervals of 3 months, 6 months, 9, months, 12 months, 18 months, 24 months, 30 months, 36 months, 42 months, 48 months after the date of baseline visit. The PPMI data includes a series of assessments (motor and non-motor assessments), Omics data (including DNA Genotyping, RNA Sequencing), and biofluids (plasma, serum, whole blood, urine, and saliva), general neurological and physical examination, etc. For our analysis, we use only a subset of the PPMI dataset that includes transcriptomic data, motor assessment questionnaire, MDS-UPDRS score, demographics, etc.

In PPMI, not all the feature’s data was collected for all the visits. For example, RNA-Seq is performed for only selected visits such as Baseline visit, 6-month visit, 12-month visit, 36-month visit, and 48 months visit whereas Motor Assessment questionnaires were performed for all the visits. Hence, we need to perform a merge operation of these datasets and select the samples for whom all the clinical data is available. We also created a few variables that are derived from primary features. In total, the dimension of the input features for our study is 34,682 that includes categorical and continuous data types of transcriptomic data (RNA-Sequence) and non-transcriptomic data (MDS-UPDRS Questionnaire I, II, III, IV, demographics, general exam, etc.). The target variable is the MDS-UPDRS final score that is a continuous variable. The complete list of features used for our analysis is presented in Supplementary Table S1.

For our analysis, only those samples for whom MDS-UPDRS-III performed in the “OFF” medication state were selected to form the disease status target variable. For a disease progression prediction problem, we need to have the motor and nonmotor condition of a subject while he/she was not on any dopaminergic medication. Also, the number of visits varies with patients in the dataset with an average number of 3.2 visits per patient. The maximum number of visits available is 5 visits and the minimum number of visits available is 1 visit. The detailed sample selection criteria for our analysis are presented in Supplementary Table S2. The criteria yielded in a data subset of 423 PD subjects with a variable number of visits and 34,682 predictor variables.

### Preprocessing steps

In the PPMI RNA-Sequence resource, the abundance estimates of RNA transcripts are available as quantization files per subject and per visit. Each quantization file has rows of 34,571 target genes we extracted the estimate for Transcripts per Million value from each file for 34,571 target genes and merged it into one file to create the predictor variable dataset. The target variable was created by adding all the answers to the Questionnaire MDS-UPDRS (Part-I, II, III, IV) for a subject’s visit. The detailed preprocessing steps performed for our study are included in the Supplementary material.

### Modelling of problem statement for PD progression

It is crucial to use the RNA sequence data from numerous prior visits to forecast a patient's MDS-UDRS score for the upcoming hospital visit. In order to comprehend the patient's condition, the problem is modeled in a way that when a patient's Baseline year (the first year) RNA-seq data is given to the predictive model, it predicts the patient's MDS-UPDRS score for the next year. The model predicts the third year's MDS-UPDRS score when the baseline year's and the second year's RNA-seq data are used as input, and the fourth year's MDS-UPDRS score when the baseline year's, second year's, and third year's data are used as input. In order to estimate the disease status in the near future, the model takes advantage of the temporal trends in the historical RNA sequence data as we advance through time. This strategy has been previously used for disease progression in Alzheimer’s Disease^13^.

Given there are N annual visits for a subject, the input at time t_<n>_ where n = 1, 2, 3...N is a feature vector denoted by X_<n>_. The feature vector X_<n>_ has a Q number of features that combine both RNA-Seq features and non-RNA features represented at the nth visit of the patient. For example, X_<1>_ is a feature vector with Q features at the first visit of the patient. Further, if the “ < N > ^”^ visit represents the current visit of the subject, then the “ < N + 1 > ” visit represents the immediate next year’s visit. The MDS-UPDRS final score at the Nth is represented by **y**_**<n>**_ and the future value of MDS-UPDRS is represented by **y**_**<n+1>**,_ then the proposed predictive model in its most basic form can be written as follows:

1 $$ \hat{y}_{ < n + 1 > } = {\text{f}}\left( {{\text{X}}_{{ < {1} > ,}} {\text{X}}_{{ < {2} > ,}} {\text{X}}_{{ < {3} > , \ldots }} {\text{X}}_{{ < {\text{n}} > }} } \right) $$

where the output $\hat{y}_{ < n + 1 > }$ is the predicted value for the MDS-UPDRS score of the next visit of the patient and the function f(.) represents the core block of our proposed model. The Eq. (1) is modified further to include the ground-truth value of MDS-UPDRS scores of the current and previous visits. For example, to predict the MDS-UPDRS score of (N + 1)th visit, we input the model with the MDS-UPDRS score of Nth visits and all the previous visits (N-1)th, (N-2)th,…., 1st. Further, we add the time interval between two consecutive visits. Let the time interval between the first visit and the immediate second visit be denoted by ∆t_<1>_ then the time interval between Nth visit and (N + 1)th can be denoted by ∆t_<n>_. The final version of our proposed model is as follows:

2 $$ \begin{aligned} \hat{y}_{ < n + 1 > } = & {\text{f}}({\text{X}}_{{ < {1} > ,}} {\text{X}}_{{ < {2} > ,}} {\text{X}}_{{ < {3} > , \ldots }} {\text{X}}_{{ < {\text{n}} > }} ; \\ & \Delta {\text{t}}_{{ < {1} > ,}} \Delta {\text{t}}_{{ < {2} > ,}} \Delta {\text{t}}_{{ < {3} > , \ldots }} \Delta {\text{t}}_{{ < {\text{n}} > }} ; \\ & {\text{y}}_{{ < {1} > }} ,{\text{ y}}_{{ < {2} > }} ,{\text{ y}}_{{ < {3} > }} , \ldots {\text{ y}}_{{ < {\text{n}} > }} ) \\ \end{aligned} $$

**Figure 1:**
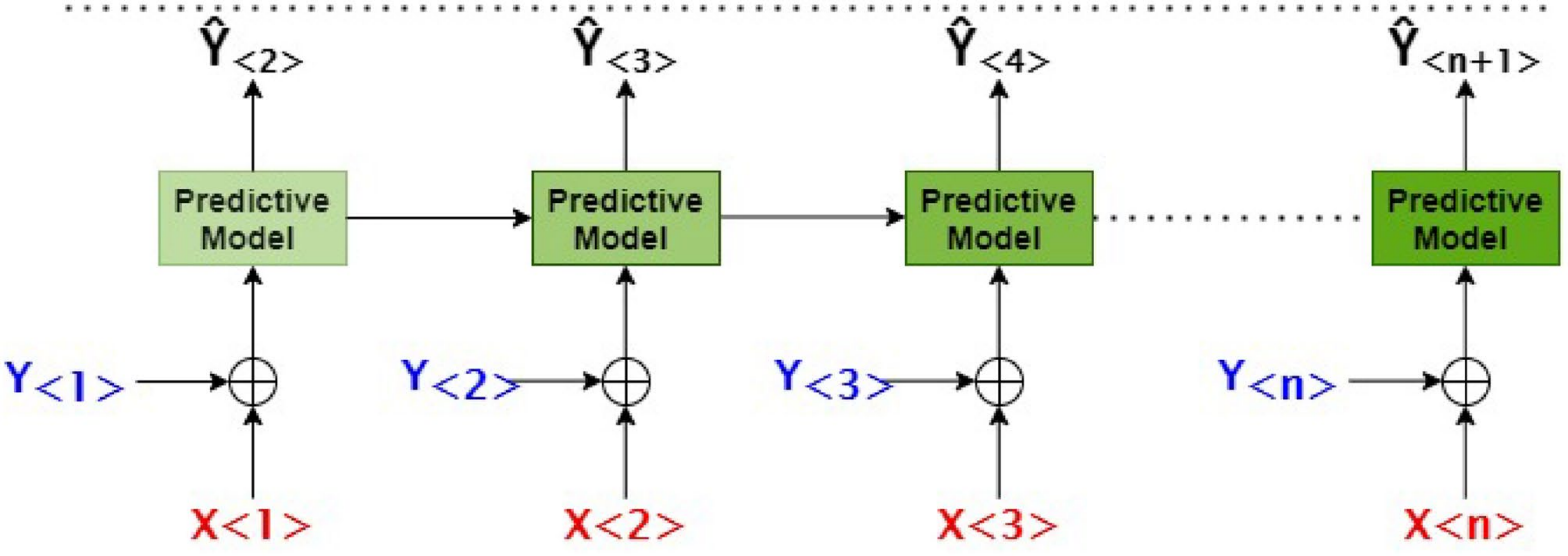
Block diagram of the problem statement.

The model in Eq. (2) can be represented pictorially in Fig. 1

### RNN model for PD progression

**Figure 2:**
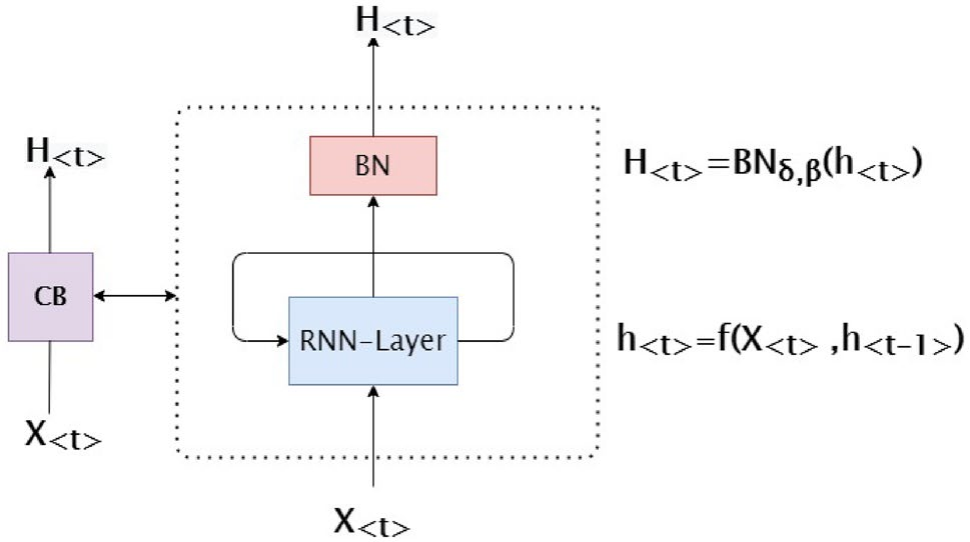
Composite Block (CB). The block comprises an RNN layer followed by a Batch Normalization (BN) layer. The RNN layer in the CB block has several hidden RNN cells, we identify the number of hidden RNN cells in the RNN layer as an important hyperparameter that is needed to be tuned.

The novelty of our proposed architecture is in combining RNN with the recent advancements done on Convolution Neural Networks (CNN) such as dense connections^16^ and batch normalization^17^. The introduction of dense connections showed several advantages in CNNs such as strengthening feature propagation, reduction in the number of parameters in the model, facilitating training with fewer data samples and encouraging feature reuse^16–19^. Additionally, batch normalization led to the speed up of the training and allowed the networks to generalize better by reducing the internal covariate shift. Though batch normalization was primarily introduced in CNNs, it was also employed into RNNs in the context of language modeling, question answering^18^, sentiment classification^19^. Motivated by the above improvements in CNNs and considering the nature of genetics data that is characterized by fewer data samples, huge feature size, and variable-length visits of patients, we hypothesize that the densely connected batch normalized RNNs may help in learning granular details from RNA-Seq data for PD disease progression. To the best of our knowledge, the densely connected RNNs are not employed in the disease progression of PD. Motivated by DenseNet^16^, we propose a densely connected RNN architecture that is composed of multiple Dense Blocks (DBs) stacked on top of each other such that the input of every DB is connected to the output of every other DB in a feed-forward fashion. The output of the last DB is connected to a fully connected neural network. Furthermore, each DB is composed of multiple Composite Blocks (CBs) stacked on top of each other such that the input of every CB is connected to the output of every other CB in a feed-forward fashion. To this end, a CB is a composite layer that contains an RNN layer followed by a Batch normalization layer. Let us understand each of the above segments in detail in the following subsections:

Composite RNN Blocks (CB): Motivated by^20^, we define ${\varvec{H}}_{\left\langle t \right\rangle } \left( . \right)$ as a composite block containing an RNN layer followed by a batch normalization layer. We apply batch normalization to the output activations of the RNN layer at time step *t*. The output of the composite block ${\varvec{H}}_{\left\langle t \right\rangle }$ at time step *t* can be written as follows:

3 $$ {\varvec{H}}_{\left\langle t \right\rangle } = BN_{\beta ,\gamma } \left( {{\varvec{h}}_{\left\langle t \right\rangle } } \right) $$

where

4 $$ BN_{\gamma ,\beta } \left( {\varvec{h}} \right) = \beta + \gamma \odot \frac{{{\varvec{h}} - E\left[ {\hat{\mathbf{h}}} \right]}}{{\sqrt {Var\left[ {\hat{\mathbf{h}}} \right] + \varepsilon } }} $$

5 $$ {\varvec{h}}_{ < t > } = f\left( {{\varvec{x}}_{ < t > } ,{\varvec{h}}_{ < t - 1 > } } \right) $$

here $\gamma {\text{and}} \beta$ are the model parameters that will be learned using backpropagation. $E\left[ {\hat{\mathbf{h}}} \right]$ and $Var\left[ {\hat{\mathbf{h}}} \right]$ are the sample mean and variance that is estimated on the current mini-batch. ${\varvec{x}}_{ < t > }$ is the vector input at the current time step *t* whereas ${\varvec{h}}_{ < t - 1 > }$ is the vector output of the cell state of the same RNN hidden layer from the previous time step. The function $f\left( . \right)$ depends upon the type of the cell in the RNN layer viz. LSTM, Gated Recurrent Units (GRU), or Vanilla. A simple CB may be represented in Fig. 2.

**Figure 3:**
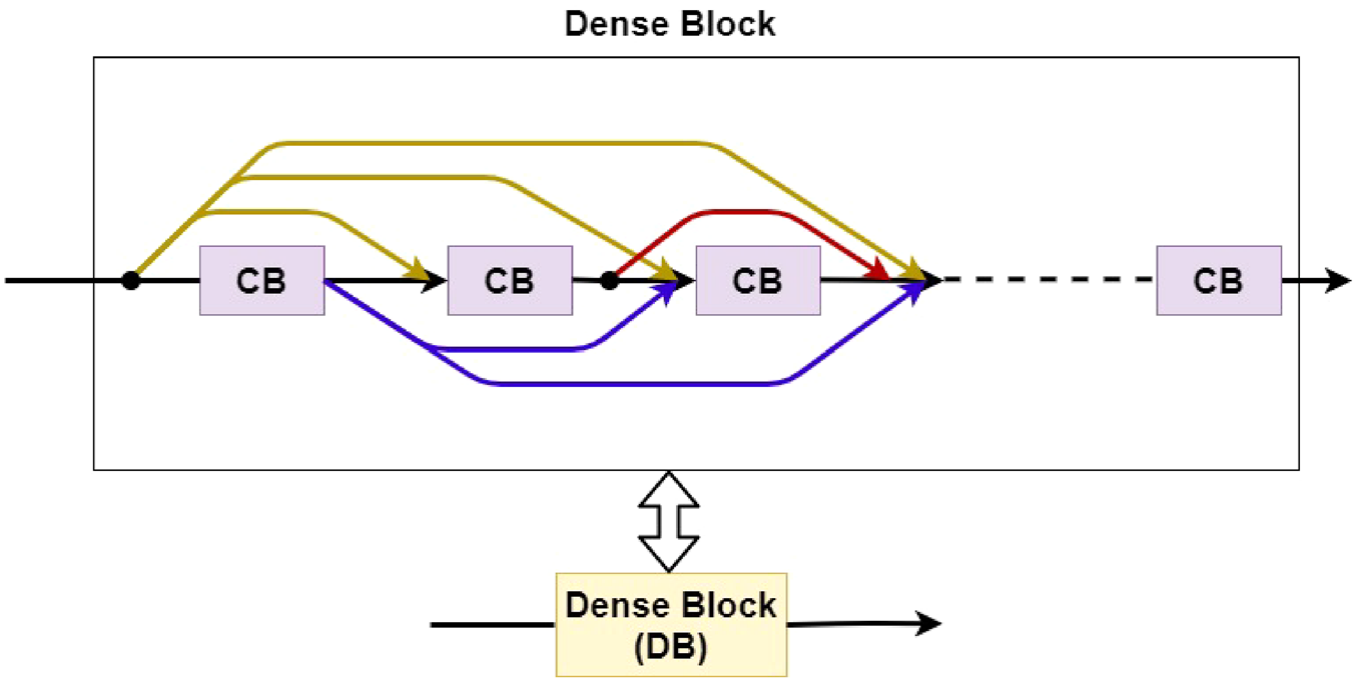
Dense Block (DB). Multiple composite blocks are stacked on top of each other and are connected in a dense fashion. The number of CBs in a dense block is identified to be an important hyperparameter. It is not necessary that all the composite blocks have an equal number of RNN cells.

Dense Connectivity of several CBs: Multiple CBs are stacked on top of each other such that the input of every CB is connected to the output of every other CB in a feed-forward fashion to form one Dense Block (DB). The connection is defined by a concatenation operator. A densely connected CB can be shown in Fig. 3.

**Figure 4:**
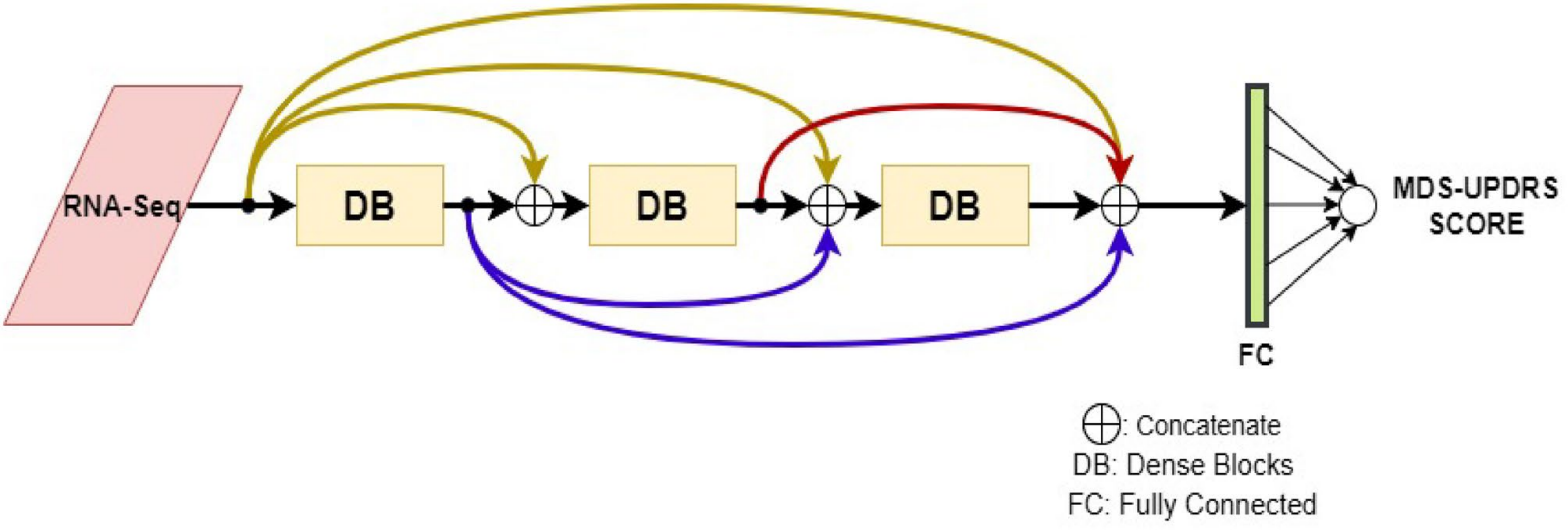
Proposed Densely Connected RNN Architecture. Multiple Dense blocks are stacked on top of each other and are connected in dense fashion. The output of the last dense block flattened and connected to a single neuron. Note that the number of dense blocks is an important hyperparameter and may be tuned.

Dense Connectivity of several DBs: Multiple DBs are stacked on top of each other such that the input of every DB is connected to the output of every other DB in a feed-forward fashion to form the proposed Densely connected RNN architecture for PD progression using RNA-Sequence data. An architecture with 3 Dense Blocks is shown in Fig. 4.

### Evaluation metrics

We know that PD is a progressive disease and usually the disease status of the patient will either worsen by next year’s visits or would remain the same. In rare cases, the disease status would become better, but we know this will not happen until an effective treatment is given to the patient which is currently not available. To a patient, he/she might be concerned with how accurate the predicted value of the future year’s MDS-UPDRS score is. We know that the model can be utilized such that when RNA-Seq of the 1st visit is given as input to the model, it predicts the 2nd year’s disease status score. And when the first year’s and second’s RNA-Seq information is given as inputs, it predicts the 3rd year’s visit disease status score. Likewise, to predict the 4th year’s visit disease status, the model takes the past information of 1st, 2nd, and 3rd year and the 5th year prediction follows the same pattern. The RMSE produced by the model in predicting the MDS-UPDRS score for all the prediction time points is averaged to give the metric called Progression Identification Error (PIE). In addition, the rank order correlation between the actual and predicted MDS-UPDRS is averaged for all prediction time points and is defined as a Progression Identification Correlation (PIC). A mathematical definition of the PIE and PIC is given in the Supplementary material.

To assess if the model can properly predict MDS-UPDRS score as well as whether providing the model with data from multiple historical visits has an impact on the model's capacity to catch trends from past visits. Let us understand this evaluation scheme with an example. Consider a patient with a total number N visits. For predicting MDS-UPDRS for the (N + 1)th visit, the model may be provided with only most recent visit’s data i.e., at Nth time point. In this case, the data at (N-1)th, (N-2)th,…,2nd,1st visits are not given to the model and the RMSE say RMSE_<N>_ for such a prediction is noted. In the second case, to predict the same (N + 1)th disease status, we now provide data from the past two visits at Nth and (N-1)th and the RMSE_<N,N-1>_ is noted. In the third case, to predict the disease status at (N + 1)th , we provide the data from last three visits i.e., at Nth, (N-1)th and (N-2)th and the RMSE_<N,N-1,N-2>_ is noted. Similarly, we note RMSE_<N,N-1,N-2, N-3>_ and proceed to find further RMSEs until we reach RMSE_<N,N-1,N-2, N-3,…0.1>_ that is calculated by the model that is given the data from all the historical visits.

## Results

In this study, we implemented our proposed densely connected RNN model with 256 Vanilla RNN cells in each composite block. A total of 4 such composite blocks form one dense block. The network is composed of 3 such dense blocks. The loss function was a mean square error and the Nadam optimizer was used in the loss function optimizer^21^. L2 regularization was applied on weights in the RNN layers. A learning rate schedule was employed to reduce the learning rate by a factor of 1/5 for every 10 epochs if there is no improvement in the validation loss. The training happened in a mini-batch fashion with a batch size of 16 subjects where each subject had the same number of time sequence/visits data. The architecture is named as “*Dense Vanilla*”.

We performed fivefold Cross-Validation (CV) on the test dataset to do the comparison in performance between the proposed model and the baseline models, and the model Dense-Vanilla achieved an RMSE of (mean = 6.01, standard deviation = 0.41) in predicting the MDS-UPDRS score and showed a rank order Correlation of (mean = 0.83, standard deviation = 0.02, *p* < 0.0001) between the predicted MDS-UPDRS and the true MDS-UPDRS. The metrics are reported in mean and standard errors in Table 1. We compare the performance of the proposed model with the performance of classical machine learning techniques including Linear Regression (LR), Support Vector Machines (SVM), Decision Trees (DT), and Random Forest (RF). These methods has been previously used for predictive modeling of AD progression^13^ and thus can be safely adopted for PD progression. We adopt a commonly used training strategy for aggregating features of all patients’ historical visits to train these baseline models^13,22^. That is, numerical, ordinal and nominal features across different visits are aggregated respectively with their corresponding mean, median and mode computed over all historical N visits. Details of this step is provided in the supplementary material.

**Table 1:** Performance comparison between our proposed dense vanilla model and baseline methods.

Models	Evaluation metrics	
	PIE (RMSE)	PIC (Correlation)
Our model (Dense-Vanilla)	6.01 ± 0.185	0.83 ± 0.01
Linear regression	12.9 ± 0.279	0.38 ± 0.02
SVM	9.87 ± 0.214	0.37 ± 0.05
Decision trees	8.39 ± 0.469	0.68 ± 0.03
Random forest	8.12 ± 0.286	0.723 ± 0.024

**Figure 5:**
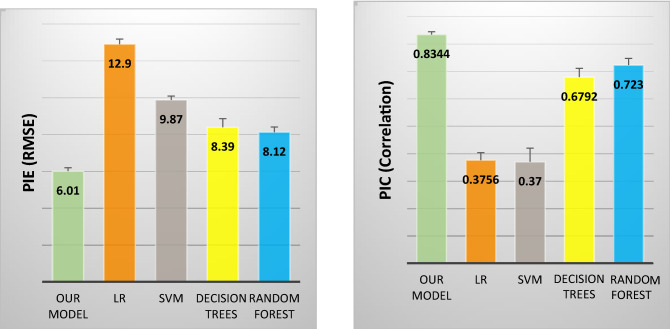
Comparison of Performance.

To test if the improvement in the performance of our proposed model was statistically significant as shown in Fig. 5 as compared to the comparison methods, the metrics obtained from 5-Fold CV were given to the student’s t-test with a significance level of alpha = 0.05, degrees of freedom = 4. The results are described in Table 2.

**Table 2:** Statistical significance test for model comparison.

Models	Hypothesis testing	
	t-Test on PIE(RMSE)	t-Test on PIC(Correlation)
Our model and linear regression	t-stat = − 20.58, *p* = 0.000	t-stat = − 18.9, *p* = 0
Our model and SVM	t-stat = − 13.65, *p* = 0.0001	t-stat = − 9.07, *p* = 0.0004
Our model and decision trees	t-stat = − 4.72, *p* = 0.0046	t-stat = − 4.5,*p* = 0.0054
Our model and random forests	t-stat = − 6.19, *p* = 0.0018	t-stat = − 4.11, *p* = 0.0074

**Figure 6:**
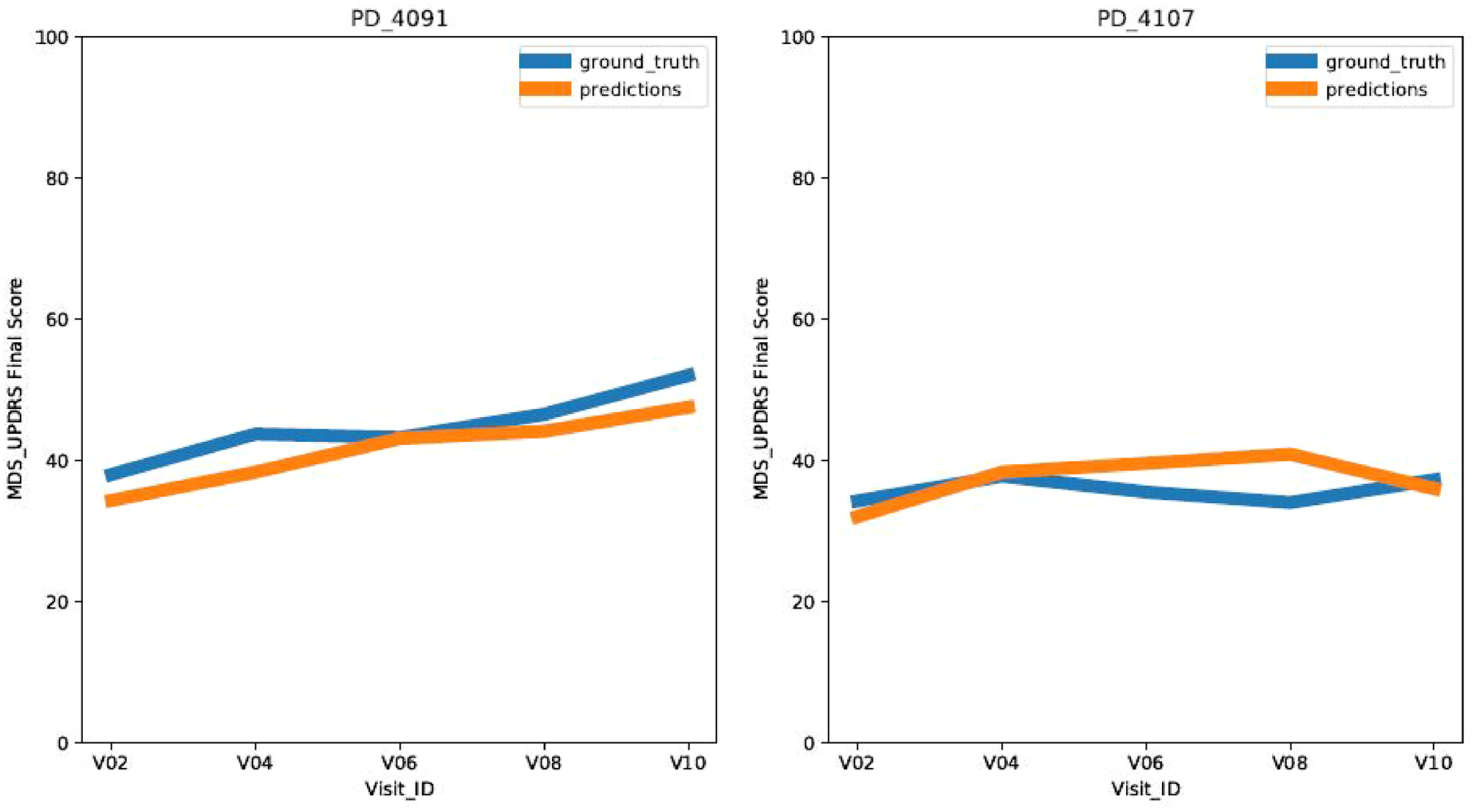
Predicted and Ground Truth disease progression curves for Test Patients. Each subplot belongs to one test patient. The curve in blue is the ground truth curve and the curve in orange is the one predicted by our Predictive Model. The x-axis has discrete visit points of 6 months(V02), 12 months (V04), 24 months (V06), 36 months (V08), 48 months (V10) and the y-axis has the MDS-UPDRS score for the patient.

In addition, to analyze the impact of various feature categories on the model performance, we conducted experiments with and without non-transcriptomic feature categories (Motor Assessment, Subject Characteristics, General Exam, Age, Time Interval) and the results are provided in Table 3 and (Fig. 6).

**Table 3:** Results of the proposed dense vanilla model with and without non-transcriptomic input features.

Feature categories	Evaluation metrics	
	PIE (RMSE)	PIC (Correlation)
RNA-Seq with non-transcriptomic features	6.01 ± 0.185	0.83 ± 0.01
RNA-Seq features only	6.87 ± 0.265	0.71 ± 0.03

### Effect of dense connections

We would like to see the performance of the architecture by changing the RNN cell to GRU and LSTM, keeping all the other hyperparameters the same. Furthermore, we compare the performance of densely connected RNN models with that of models with plain configurations. Here plain configuration means that no dense connections were introduced either between Composite Blocks or between Dense Blocks. All the other hyperparameters (structural, optimization, and regularization) were kept the same to provide a fairground for comparison. As shown in Table 4, Dense Connections consistently provide improvements for different types of RNN cells.

**Table 4:** Performance comparison between densely connected RNN models and plain RNN configuration.

RNN cell type	Model configuration	Evaluation metrics	
		PIE (RMSE)	PIC (Correlation)
Vanilla RNN	With dense connections	6.01 ± 0.185	0.83 ± 0.01
	Without dense connections	11.428 ± 0.525	0.06 ± 0.038
GRU	With dense connections	6.22 ± 0.344	0.81 ± 0.016
	Without dense connections	9.894 ± 0.784	0.59 ± 0.033
LSTM	With dense connections	6.22 ± 0.124	0.84 ± 0.010
	Without dense connections	13.251 ± 0.603	0.53 ± 0.032

As illustrated in Table , there is a significant difference in the RMSE obtained by models with dense Connections and models without dense Connections. We observe the behavior for all three categories of RNN cell types (Vanilla RNN, GRU, and LSTM). In each of the categories, the model with dense connections has better RMSE in predicting the MDS-UPDRS as compared to the model without dense connections. Furthermore, a higher rank order correlation in predicting disease status is obtained when the models has dense connections.

### Effect of batch normalization

We observed that the proposed composite function block CB plays an important role in helping the densely connected RNN models to learn. The Batch Normalization layer on top of the RNN layer in a CB block is integral to the successful learning of our models. We conducted experiments where the normalization layer in the CB block was removed and the remaining model configurations remained the same. Table 5 shows the performance comparison between the models that are identical to each other in all aspects except the batch normalization layer in the CB block.

**Table 5:** Performance comparison between densely connected RNN models with and without batch normalization.

Densely connected RNN models	Model configuration	Evaluation metrics	
		PIE (RMSE)	PIC (Correlation)
Vanilla RNN-dense connections	With batch normalization in CB	6.01 ± 0.185	0.83 ± 0.01
	Without batch normalization in CB	18.315 ± 5.323	0.40 ± 0.086
GRU-dense connections	With batch normalization in CB	6.22 ± 0.344	0.81 ± 0.016
	Without batch normalization in CB	11.491 ± 1.801	0.40 ± 0.096
LSTM-dense connections	With batch normalization in CB	6.22 ± 0.124	0.84 ± 0.010
	Without batch normalization in CB	11.253 ± 1.755	0.38 ± 0.099

In each of the categories of RNN cell types (Vanilla RNN, GRU, and LSTM), the model with batch normalization has lower RMSE in predicting the MDS-UPDRS as compared to the model without batch normalization. Furthermore, a higher rank order correlation in predicting disease status is obtained when the models have batch normalization.

### Ethical approval

This study was funded by the NSERC CREATE funded “Biomedical Engineering Smartphone Training” program (CREATE-BEST), and the Office of Research Ethics and Integrity at the University of Ottawa reviewed and approved.

## Discussion

As illustrated in Figure our model is able to capture the trend and predict the MDS-UPDRS scores for a period of 4 years at discrete visit points of V02 (6 months), V04 (12 months), V06 (24 months), V08 (36 months), and V10 (48 months). For the sake of simplicity, we presented the disease progression graphs for the patients in the test dataset with 5 visits.

For almost all the test patients, the disease progression predicted by our model aligns with the ground truth disease progression values. This confirms that our proposed model was able to learn the medical patterns and granular features from the longitudinal data of the transcriptome of the patient. As per the 5-CV model evaluation, we know that the predictive model has an RMSE of 6.01 in predicting the MDS-UPDRS score and a rank correlation of (PIC = 0.83, *p*-value = 0.0001).

As illustrated in Table , there is a significant difference in the RMSE obtained by our proposed model as compared to the classical machine learning methods of Linear Regression, SVM, Decision Trees, and Random Forests. Thus, our model outperforms all the baseline methods in terms of RMSE and PIC in predicting the MDS-UPDRS score. Furthermore, there is a significant difference in the correlation obtained by our proposed model as compared to the baseline methods. As illustrated in Table , the improvements in the performance of our model as compared to the performance of baseline methods are statistically significant at a significance level of 0.05.

As indicated in Table , we wanted to know the impact of adding non-transcriptomic input features such as Motor Assessment, Subject Characteristics, General Exam, Age, Time Interval to the RNA-Seq features while training the proposed model. It will be interesting to discuss the acceptability of RMSE of 6.01 in real-world applications. While MDS-UPDRS is a highly valid and reliable instrument to capture the true phenotype^23,24^, it is susceptible to “noise”^25^. The two major sources of noise are measurement error (inter and intra-rater variability) and short-term effects (mood, stress, climate, time of the day, etc.) that are irrelevant to the overall progression of the disease^24^. It is reasonable to state that if the RMSE of 6.01 in predicting the MDS-UPDRS is lesser than or equal to the noise inherent in estimating the true MDS-UPDRS score then the predictive model has an acceptable error for real-world applications.

To this end, as per the study^25^, the authors calculated the error variance due to noise in estimating the true value of MDS-UPDRS score and reported an error variance of 4.87 in an estimating score of part-I, 3.66 in part-II, and 15.52 in Part-III of MDS-UPDRS. The total noise component of the MDS-UPDRS score (part-I, part-II, and part-III) is thus 8.059. As the RMSE of 6.01 in predicting the total MDS-UPDRS score by our predictive model is lesser than the inherent noise of 8.059 in the instrument itself, it is safe to assume that the performance of the predictive model is acceptable for real-world applications. Moreover, as per the recent study^26,27^ in predictive modeling of PD using imaging genetics on a combination of DNA genotyping and neuroimaging, the authors reported an RMSE of 7.82 in predicting MDS-UPDRS-Score. This value of RMSE is comparable to the RMSE of our predictive model that uses transcriptomic data.

## Conclusion

To the best of our knowledge, this study is the first to investigate the usefulness of Omics data such as RNA-Seq in the predictive modeling of the PD Disease Progression using densely connected deep RNNs.

The contributions of this study are as follows:

1. We proposed a deep RNN structure that can predict the future year’s MDS-UPDRS score of a patient by taking the inputs of the previous year’s RNA-Sequence data. The model can leverage the temporal patterns in the historical RNA sequence data.
2. The Predictive model is adaptive over time as the model was trained with irregular visit time intervals and the various number of visits. The model is able to predict the MDS-UPDRS score with an RMSE of 6.01 and a rank correlation of 0.83 between the predicted and true values of MDS-UPDRS.
3. We observed that the introduction of Batch Normalization and Dense Connections play an important role in making the multi-layered RNN to learn the features from high dimensional gene expression data.

## Supplementary Information

Supplementary Information.

## Data Availability

The datasets are available to download from the PPMI on the date: 2018-06-05 and the latest update of all datasets are downloaded on 2020-01-28. In addition, the corresponding author will provide the dataset utilized and/or analyzed for the current work upon reasonable request.
