# Supplementary material for: Predictive modelling of Parkinson’s disease progression based on RNA-Sequence with densely connected deep recurrent neural networks
Source: Sci Rep. 2022 Dec 12;12:21469. doi: 10.1038/s41598-022-25454-1 (PMC9744878; doi:10.1038/s41598-022-25454-1)
Supplement: Supplementary file 1 — Supplementary Information. [file 41598_2022_25454_MOESM1_ESM.docx]

**Supplementary document**

**Predictive Modelling of Parkinson’s Disease Progression Based on RNA-Sequence with Densely Connected Deep Recurrent Neural Networks**

Siraj Ahmed^1^, Majid Komeili^2^*_,_ and Jeongwon Park^1,3^*

^1^School of Electrical Engineering and Computer Science, University of Ottawa, Ottawa, ON, K1N 6N5, Canada

^2^School of Computer Science, Carleton University, Ottawa, ON, K1S 5B6, Canada

**^3^**Department of Electrical and Biomedical Engineering, University of Nevada, Reno, NV 89557, USA

*Email: MajidKomeili@cunet.carleton.ca, [jepark@unr.edu](mailto:jepark@unr.edu),

## Description of Datasets in PPMI database

### Description of the subjects under study in PPMI:

The PPMI study has enrolled 423 PD subjects till now who were drug naïve (i.e. not much treated with dopaminergic medications) and who have been diagnosed with PD for a period of less than 2 years at the time of enrollment into the study. This is one of the most important differences in PPMI study when compared to other studies such as PDBP or BioFind that the subjects in PPMI are all ‘early diagnosed’ or ‘de novo’. The study has about 196 healthy subjects and plans to recruit more five types of cohorts, viz: prodromal (100 individuals), patients who have scans without evidence of dopaminergic degeneration (i.e., SWEDD, 70 individuals), a genetic cohort of PD patients (600 individuals), a genetic registry (600 individuals).

### Description of the Input Features and Target Variable for our Study:

The subjects are followed up for a period of 4 years from the date of enrollment and the clinical data is collected every (3 months, 6 months, 9, months, 12 months, 18 months, 24 months, 30 months, 36 months, 42 months, 48 months) after the date of baseline visit during the follow-up period. The data collected includes a series of assessments (motor and non-motor assessments), Omics data (including DNA Genotyping, RNA Sequencing), and biofluids (plasma, serum, whole blood, urine, and saliva), general neurological and physical examination, etc. However, for our analysis, we only use a subset of the PPMI dataset that includes transcriptomic data, motor assessment questionnaire, MDS-UPDRS score, demographics, etc. We also created a few variables that are derived from primary variables. The complete list of features used for our analysis is presented in Table below. For ease of understanding, we grouped the input features into 5 groups.

**Table S1.** Description of Dataset of our Study

| Group of features | | Description of the feature/ group of features | Number of features/variables | Type of variable |
| --- | --- | --- | --- | --- |
| **Predictor Variables** | | | | |
| RNA Sequencing | Quantification file of Transcripts | Each file contains 34571 gene names. For each gene, following information is available after RNA-Seq:  -gene length  -effective length  -TPM  -NumReads  For our analysis, we use Transcripts per Million (TPM), as it is the recommended abundance estimate of the transcripts of the genes. Other estimates are discarded. | 34571 features (expressed in units of TPMs) | Continuous |
| Motor Assessment | MDS-UPDRS-I Questionnaire | This part has questions related to non-motor experiences of daily living and is self-administered by the patient | 13 | Categorical |
|  | MDS-UPDRS-II Questionnaire | This part has questions related to motor experiences of daily living is self-administered by the patient | 13 | Categorical |
|  | MDS-UPDRS-III Questionnaire | This part has questions related to motor experiences and is completed by a healthcare professional after examining the patient | 33 | Categorical |
|  | MDS-UPDRS-IV Questionnaire | This part has questions to be answered by a healthcare professional to assess two motor complications, dyskinesias and motor fluctuations that include OFF-state dystonia | 6 | Categorical |
|  | Hoehn and Yahr Stage | The “Hoehn and Yahr stage “is now a part of MDS-UPDRS-III and has 5 classes. However, was originally published in 1967 to measure the Parkinson symptoms and disability | 1 | Categorical |
| Subject Characteristics at time of screening | Demographics | Features such as birthdate, gender, and race | 11 | Categorical |
|  | Family status | Features to answer if close family relatives had PD | 18 | Categorical |
|  | Socio Economics | Features regarding the educational level of the patient | 2 | Categorical |
| General Exam | Physical Exam |  | 11 | Categorical |
|  | Vital Signs |  | 10 | Categorical and Continuous |
| Derived features | Time Interval between two visits | The difference in time between two successive visits | 1 | Continuous |
|  | Age | The Age variable is derived from “BIRTHDT” variable of Demographics page of subject. The difference between the birthdate and the visit year. | 1 | Continuous |
| **Total number of input features 34,682** | | | | |
| **Target Variable** | | | | |
| Group of features | | Description of the feature/ group of features | Number of features/variables | Type of variable |
| MDS-UPDRS Final Score | | The target variable (MDS-UPDRS) reflects the motor symptoms and clinometric properties of PD on a scale of 0 to 272 with 0 being normal and 272 being severe motor and non-motor decline | 1 | Continuous |

Table S1 gives the information of the subset of features from PPMI datasets that we use for our analysis. The target variable for our analysis is MDS-UPDRS final score which is a continuous variable whose value ranges from 0 to 272 with 0 being normal and 272 being severe motor and non-motor decline. The primary predictor variables are the gene expression levels i.e. RNA transcripts expressed in Transcripts per Million. In addition to gene expression values, we also use the basic general information of patients that was collected at screening such as demographics, family history, and socioeconomic status. we have also created two variables called “Time interval” and “Age” that are derived from the primary features. The time interval for a visit is created by the difference in time between that current visit and the immediate next visit available. The “Age” variable for a visit is the age of the patient on that visit and is created by the difference in the birth year and the year of that visit.

### Number of Samples in our study:

This section describes the sample size for our subset of the dataset. We discuss the frequency of information collection in PPMI.

The data for features described in the table (except the subject characteristics), was conducted by PPMI for all the subjects- *longitudinally* i.e. for various “visits” or “time points” during the study period of 4 years starting from screening(SC), baseline visit(BL), 3-months(V01), 6-months(V02), 9-months(V03), 12-months(V04), 18-months(V05), 24-months(V06), 30-months(V07), 36-months(V08), 42-months(V09), 48-months(V10). However, not all of the feature’s data was collected for all the visits. For example, RNA-Seq is performed for only selected visits such as Baseline visit, 6 months visit, 12 months, 36 months, and 48 months whereas Motor Assessment questionnaires were performed twice for all the visits. Hence, we need to perform a merge operation of these datasets and select the samples for whom all the clinical data is available. Table S2 below describes in detail the frequency of collection of data for each feature subset in PPMI. This information will help us to understand the data selection criteria we provide in the next subsection Data Selection Criteria

**Table S2.** Frequency of Information Collection in PPMI

| RNA-Seq Data | Performed once for annual visits: Baseline (BL), 12-months (V04), 24-months(V06), 36-months(V08). |
| --- | --- |
| MDS-UPDRS-I, II | Performed once for all visits |
| MDS-UPDRS-III | Performed for all visits.  But twice on annual visits (Bl, V04, V06, V08, V10) one with “ON” state medication and other with “OFF” state dopaminergic medication [add footnote].  **ON state:** The subject was given dopaminergic medication  **OFF state:** at-least 6 hours after the last dose of dopaminergic medication |
| MDS-UPDRS-IV | Performed once for all visits |
| General Exam | Performed once for all visits |

## Data Selection Criteria

This section provides the criteria for data selection from PPMI to build the subset of the dataset for our analysis:

1. **Data Download Date:** The datasets were downloaded from PPMI on the date: 2018-06-05 and the latest update of all datasets are downloaded on 2020-01-28. The analysis and the experiments were updated to include the changes in the dataset.
2. **Subject Categories:** The subjects belonging to Parkinson’s Disease (PD) category were only considered for the analysis. The number of PD subjects is 423. The subjects belonging to other categories such as HC, SWEDD, Genetic PD, Genetic Registry PD were discarded from our study.
3. **Transcriptomic data samples:** For our analysis, we included all the data samples for which RNA-Seq was performed. The RNA-Seq analysis yields a couple of outputs. We chose only a subset of output from RNA-Seq and created the RNA-Seq “Subset-1”.
4. **Disease status data samples:** For our analysis, only those samples for whom MDS-UPDRS-III performed in the “OFF” medication state were selected to form the disease status target variable. For a disease progression prediction problem, we need to have the motor and nonmotor condition of a subject while he/she was NOT on any dopaminergic medication. As per PPMI protocol, a delay of “6 hours” with no medicine is enough to withdraw the effect of the last dose of medicine on the patient.
5. **Data Sample ID:** Each data sample is represented by a unique key which is a combination of two fields - “PATNO” and “EVENT_ID”. The field “PATNO” defines the subject’s name that the data belongs to and the field “EVENT _ID” defines the time point or visit id of the data sample. This applies to all the datasets. We mapped all the data samples from the predictor variables, to the data samples in the MDS-UPDRS target variable with the help of the primary key on “PATNO” and “EVENT_ID”.
6. For our analysis, we need 3-Dimensional data, the first dimension is the number of PD patients i.e. 423, the second dimension is the number of visits and the third dimension is the number of predictor variables. To this end, the final dataset has 423 3-D samples with a variable number of visits or 1709 2-D samples.

**1.3** **Comparison Methods and Training**

For a patient with N visits the patient’s data can be represented in the form of a 3D matrix as follows:

|  | [1, N, X_1xQ_] | (1) |
| --- | --- | --- |

where Q is the length of the input feature vector. The data presented in equation (1) is a longitudinal temporal representation of a single patient’s data. The same is used to train the deep RNN models, however, to train the non-RNN baseline models, this data is converted into an aggregated form. The aggregated data vector also has Q features, where each value of the q^th^ feature (q=1,2,3,4,…….., Q) is aggregated over N visits depending upon the data type of the feature as summarized in S3.

**Table S3**. Aggregated Value for Different Data Types.

| **Data type of q^th^ feature** | **Aggregated Value for q^th^ feature** |
| --- | --- |
| Continuous type feature | ${q^{th}}_{aggregated}=\frac{q_{<1>}+q_{<2>}+q_{<3>}+....q_{<N>}}{N}$ |
| Ordinal type feature | ${q^{th}}_{aggregated}=Median(q_{<1>}, q_{<2>},q_{<3>},....q_{<N>})$ |
| Nominal type feature | ${q^{th}}_{aggregated}=Mode(q_{<1>}, q_{<2>},q_{<3>},....q_{<N>})$ |

If the q^th^ feature is a continuous type variable, then the aggregated value of that feature is the mean of the value of that feature over the past N visits. If the q^th^ feature is an ordinal data type, then the aggregated value is the median of the values of past N visits. Similarly, if the q^th^ feature is a nominal variable then the aggregated value of that feature is the mode of the values of that feature for all historical N visits.

Based on the aggregated values, the equation for the predictive model in equation (1), can now be written as follows:

|  | $\hat{y}$_<n+1>_ = f (${\overset{\sim}{X}}_{<1,2,....,n>}$) | (2) |
| --- | --- | --- |

where ${\overset{\sim}{X}}_{<1,2,....,n>}$ is a feature vector with Q dimensions (q=1,2,3,……., Q), where each of the q^th^ dimension is an aggregated value of the q^th^ feature for all the historical N visits.

## 1.4 Training Pipeline and Hyperparameter Tuning

During the training and hyperparameter tuning phase of RNN models, a train-validation-test split was performed on the dataset to reserve 60% subjects as training, 20% subjects as validation, and the remaining 20% subjects as hold outset. The training and hyperparameter tuning of the RNN models were performed using the training and validation datasets and the performance of the tuned model was tested on the test dataset.

We used the validation dataset to tune the hyperparameters of our RNN models using a grid search strategy on the hyperparameter search space. We categorize the tuning experiments into three categories: *Structural*, *Optimization,* and *Regularisation*. The Structural category has the hyperparameters such as the type of RNN cell, number of RNN cells, number of Densely Connected Blocks(DBs), number of Composite Function Blocks (CBs). The Optimization category has the hyperparameters such as learning rate, loss optimizers, type of loss, number of epochs and batch sizes, etc. whereas the Regularisation category has the hyperparameters that help to combat overfittings such as selection between L1, L2 and L1_L2 regularization, the regularization constant, dropout layer factors, etc. The table below summarizes the three categories of tuning experiments.

**Table S4**. Hyper Parameter Tuning Search Space

| **Purpose** | **Hyper parameter** | **Grid/Sample Space** |
| --- | --- | --- |
| Structural | Type of RNN Cell | [Vanilla RNN] |
|  | Number of RNN cells(*nb_cells*) | [32,64,128,256,512] |
|  | Number of Composite Function Blocks (*nb_CBs*) | [2,4,8,12,16,32] |
|  | Number of Densely Connected Blocks (*nb_DBs*) | [1,2,4,8,16] |
| No of Experiments in Structural Category= | | 150 |
|  | | |
| Optimization | Learning rate(lr) | lr $\in$ [0.0001, 1] with 5 steps |
|  | Loss | [Mean_square_error, Mean_absolute_error, Mean square_logarithmic_error] |
|  | Loss Optimizer | [Adam[45], Nadam[46], RMSProp[47], Adadelta[48], Adagrad[49], Adamax[45], SGD] |
|  | Number of epochs | [250] |
| No of Experiments in Optimization Category = | | 105 |
|  | | |
| Regularisation | Dropout factor | [0,0.2,0.5] |
|  | Type of Regularization | [L1, L2, L1_L2] |
|  | Regularization Applied on | [Weights, Bias, Recurrent, Activity] |
|  | Regularisation constant($\lambda$) | $\lambda$ $\in$ [0,0.1] with 5 steps |
| No of Experiments in Regularisation Category | | 50 |

While performing the hyperparameter tuning, we first tuned the RNN models for the Structural hyperparameters (No of CBs, DBs, Cells) keeping the hyperparameters of the remaining Optimization and regularisation initialized to default values as mentioned below in Table S5, we found the combination of Structural hyperparameters that gives the best performance, we then moved to tune the hyperparameters of the optimization category keeping the regularization aspects as constant and the structural hyperparameters initialized to the best combination. Lastly, the hyper parameters of regularization were tuned by initializing the hyperparameters of structure and optimization to the best combination. The pipeline for model training and hyperparameter tuning is detailed in the Table S7.

In this way, the total number of experiments run as part of our study was the “addition” of the number of experiments in Structural, the number of experiments in Optimization and the number of experiments in Regularization category. From the Table S4, the number of experiments run as part of the Structural category are the total combinations in the structural grid that is 150 experiments. Similarly, the number of experiments in Optimization and Regularization Categories are 105 and 50**,** respectively. As such, the total number of experiments totals to (150+105+50)=305 experiments.

**Table S**Error! No text of specified style in document.. Default Initialization values of Hyperparameters

|  | **Hyper parameter** | **Default Values** |
| --- | --- | --- |
| **Optimization Category** | Learning rate(lr) | Lr=0.001 |
|  | Loss | Mean_square_error |
|  | Loss Optimizer | Adam |
|  | Number of epochs | 250 |
|  |  |  |
| **Regularisation Category** | Dropout factor | 0 |
|  | Type of Regularization | L2 |
|  | Regularization Applied on | Weights |
|  | Regularisation constant($\lambda$) | $\lambda=0.01$ |

### Table S6. Algorithm : Model Training and Hyper Parameter Tuning


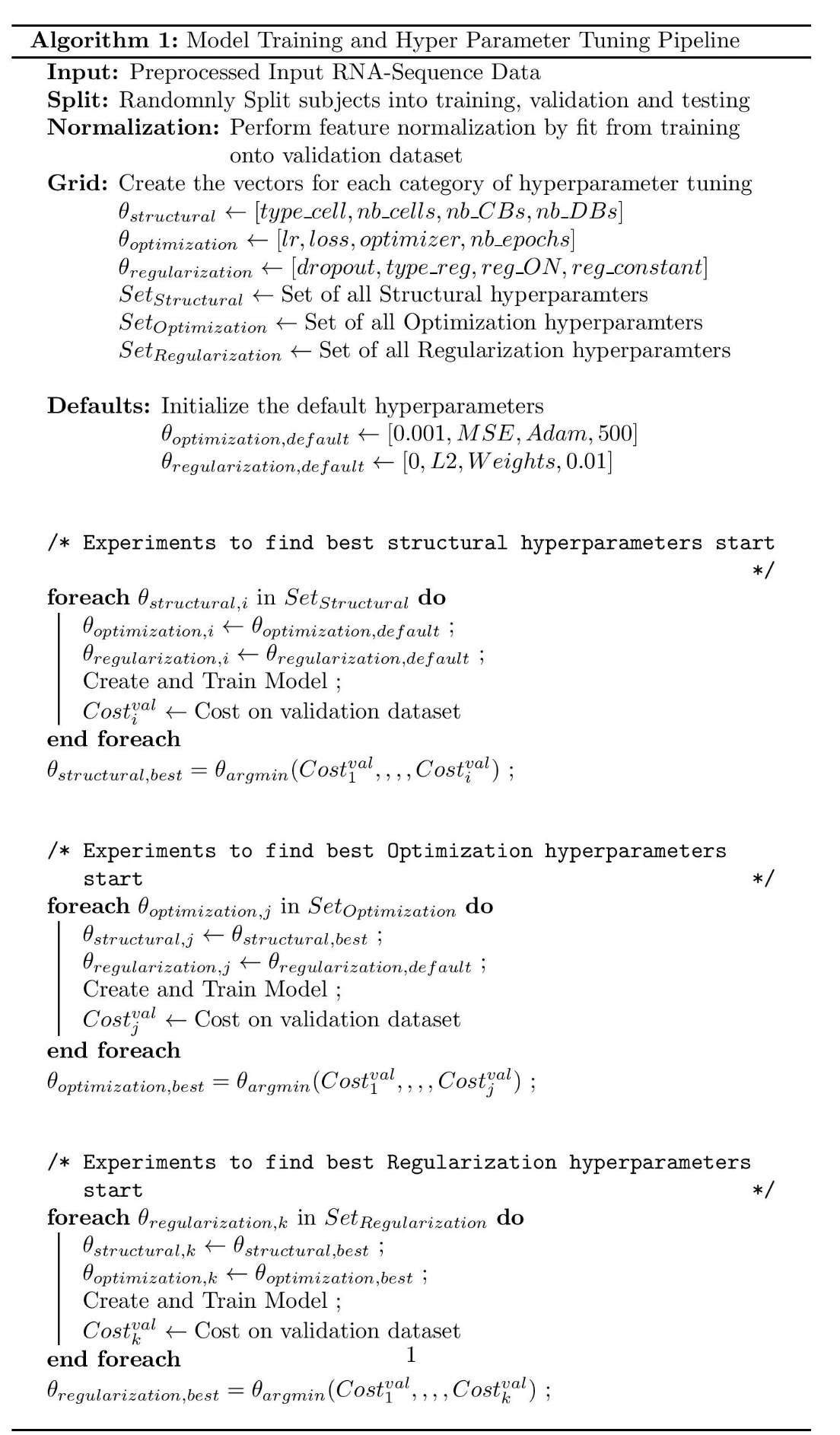


**Table S7.** List of Abbreviations

| **Abbreviation** | **Definition** |
| --- | --- |
| PD | Parkinson’s disease |
| PPMI | Parkinson Progression Marker Initiative |
| RNN | Recurrent Neural Network |
| RMSE | Root Mean Square Error |
| RNA | Ribonucleic acid |
| AI | Artificial Intelligence |
| ML | Machine learning |
| DL | Deep Learning |
| LSTM | Long Short Term Memory |
| AD | Alzheimer’s Disease |
| MDS-UPDRS | Movement Disorder Society-sponsored unified Parkinson’s disease rating scale |
| CNN | Convolution Neural Networks |
| DB | Dense Block |
| CB | Composite Block |
| GRU | Gated Recurrent Unit |
| BN | Batch Normalization |
| PIE | Progression Identification Error |
| PIC | Progression Identification Correlation |
| LR | Linear Regression |
| SVM | Support Vector Machines |
| DT | Decision Trees |
| RF | Random Forest |
| CV | Cross validation |
| V | Visit |
| NSERC | Natural Sciences and Engineering Research Council of Canada |
| CREATE | Collaborative Research and Training Experience |
| BEST | Biomedical Engineering Smartphone Training |
